# CsrA-Mediated Translational Activation of the *hmsE* mRNA Enhances HmsD-Dependent C-di-GMP-Enabled Biofilm Production in Yersinia pestis

**DOI:** 10.1128/jb.00105-23

**Published:** 2023-05-16

**Authors:** Amelia R. Silva-Rohwer, Kiara Held, Helen Yakhnin, Paul Babitzke, Viveka Vadyvaloo

**Affiliations:** a Paul G. Allen School for Global Health, Washington State University, Pullman, Washington, USA; b Department of Biochemistry and Molecular Biology, Center for RNA Molecular Biology, Pennsylvania State University, University Park, Pennsylvania, USA; NCBI, NLM, National Institutes of Health

**Keywords:** *Yersinia pestis*, CsrA, *hmsCDE* operon, biofilm production, cyclic-di-GMP

## Abstract

The plague bacterium, Yersinia pestis, forms a biofilm-mediated blockage in the flea foregut that enhances its transmission by fleabite. Biofilm formation is positively controlled by cyclic di-GMP (c-di-GMP), which is synthesized by the diguanylate cyclases (DGC), HmsD and HmsT. While HmsD primarily promotes biofilm-mediated blockage of fleas, HmsT plays a more minor role in this process. HmsD is a component of the HmsCDE tripartite signaling system. HmsC and HmsE posttranslationally inhibit or activate HmsD, respectively. HmsT-dependent c-di-GMP levels and biofilm formation are positively regulated by the RNA-binding protein CsrA. In this study we determined whether CsrA positively regulates HmsD-dependent biofilm formation through interactions with the *hmsE* mRNA. Gel mobility shift assays determined that CsrA binds specifically to the *hmsE* transcript. RNase T1 footprint assays identified a single CsrA binding site and CsrA-induced structural changes in the *hmsE* leader region. Translational activation of the *hmsE* mRNA was confirmed *in vivo* using plasmid-encoded inducible translational fusion reporters and by HmsE protein expression studies. Furthermore, mutation of the CsrA binding site in the *hmsE* transcript significantly reduced HmsD-dependent biofilm formation. These results suggest that CsrA binding leads to structural changes in the *hmsE* mRNA that enhance its translation to enable increased HmsD-dependent biofilm formation. Given the requisite function of HmsD in biofilm-mediated flea blockage, this CsrA-dependent increase in HmsD activity underscores that complex and conditionally defined modulation of c-di-GMP synthesis within the flea gut is required for Y. pestis transmission.

**IMPORTANCE** Mutations enhancing c-di-GMP biosynthesis drove the evolution of Y. pestis to flea-borne transmissibility. c-di-GMP-dependent biofilm-mediated blockage of the flea foregut enables regurgitative transmission of Y. pestis by fleabite. The Y. pestis diguanylate cyclases (DGC), HmsT and HmsD, which synthesize c-di-GMP, play significant roles in transmission. Several regulatory proteins involved in environmental sensing, as well as signal transduction and response regulation, tightly control DGC function. An example is CsrA, a global posttranscriptional regulator that modulates carbon metabolism and biofilm formation. CsrA integrates alternative carbon usage metabolism cues to activate c-di-GMP biosynthesis through HmsT. Here, we demonstrated that CsrA additionally activates *hmsE* translation to promote c-di-GMP biosynthesis through HmsD. This emphasizes that a highly evolved regulatory network controls c-di-GMP synthesis and Y. pestis transmission.

## INTRODUCTION

Yersinia pestis is the flea-transmitted bacterial agent of bubonic plague. During infection of the flea host, the pathogen colonizes the flea digestive tract and obtains nutrients from the blood meal and partially digested blood to form a cohesive biofilm mass (1–3). This biofilm facilitates a blockage in the flea foregut that is essential for regurgitative transmission of Y. pestis by fleabite (4, 5). The Y. pestis biofilm is synthesized by the gene products of the *hmsHFRS* operon (6). In numerous bacteria (7), including Y. pestis, biofilm is activated by elevated c-di-GMP levels (8, 9). In Y. pestis, c-di-GMP is synthesized by diguanylate cyclase (DGC) enzymes HmsT and HmsD (8–11).

HmsT is the predominant DGC for *in vitro* biofilms, as an *hmsT* mutant is notably deficient at producing biofilm *in vitro*. In contrast, an *hmsD* mutant exhibits a negligible reduction in biofilm production under similar culture conditions (9, 12). However, biofilm-mediated flea blockage is reduced by ~80% and 50% during *hmsD* and *hmsT* mutant flea infection, respectively, indicating that HmsD is predominantly required for biofilm formation *in vivo* (9). The *hmsD* gene is cotranscribed with *hmsC* and *hmsE* as part of the *hmsCDE* operon. HmsCDE functions as a tripartite signaling system to control c-di-GMP synthesis in response to specific environmental stimuli (12–14). Our current understanding is that the allosteric interaction between HmsC and the periplasmic domain of HmsD represses HmsD activity (12), resulting in decreased levels of c-di-GMP and biofilm production. Conversely, HmsE binds directly to HmsC to liberate HmsD from its inhibitory interaction with HmsC (13), which leads to elevated c-di-GMP levels and biofilm production. An HmsE mutant is defective in flea foregut blockage (13). As such, mechanisms that support disproportionately higher levels of HmsE, which can outcompete the HmsC-HmsD interaction, are predicted to mediate HmsD-dependent biofilm production (13).

CsrA is a highly conserved global RNA binding protein in *Gammaproteobacteria* that positively regulates c-di-GMP levels and biofilm production in Y. pestis (15, 16). CsrA controls gene expression by repressing or activating translation or by promoting transcription termination of target transcripts (17–19). CsrA binding sites in 5′ leader regions of target transcripts contain highly conserved GGA motifs. A common mechanism of CsrA-mediated translational repression involves CsrA binding to a GGA motif that overlaps the cognate Shine-Dalgarno (SD) sequence or the initially translated region, thus competing with ribosome binding (18, 19). In Y. pestis CsrA binds to a GGA motif that overlaps the *hfq* SD sequence to repress Hfq expression and alleviate Hfq-dependent repression of HmsT expression. This regulatory event leads to increases in c-di-GMP levels and, together with reduced biofilm-mediated flea foregut blockage exhibited by a *csrA* mutant (15), implicates CsrA in mediating transmission of Y. pestis by fleabite.

Regulation of c-di-GMP levels and biofilm production by CsrA occurs through multiple mechanisms in other bacterial species (20–24). For example, in Escherichia coli, CsrA directly represses translation of two mRNAs encoding DGCs, *ydeH* and *ydeT*, and indirectly inhibits expression of five other DGCs to decrease c-di-GMP levels (24). In this study we tested the hypothesis that CsrA increases HmsD-mediated biofilm production in Y. pestis by activating translation of the *hmsE* mRNA. Our results demonstrated that CsrA binds specifically to the 5′ leader region of the *hmsE* mRNA and likely alters its structure, leading to increased HmsE expression and HmsD-dependent biofilm formation.

## RESULTS

### CsrA binds specifically to the *hmsE* leader transcript.

**FIG 1:**
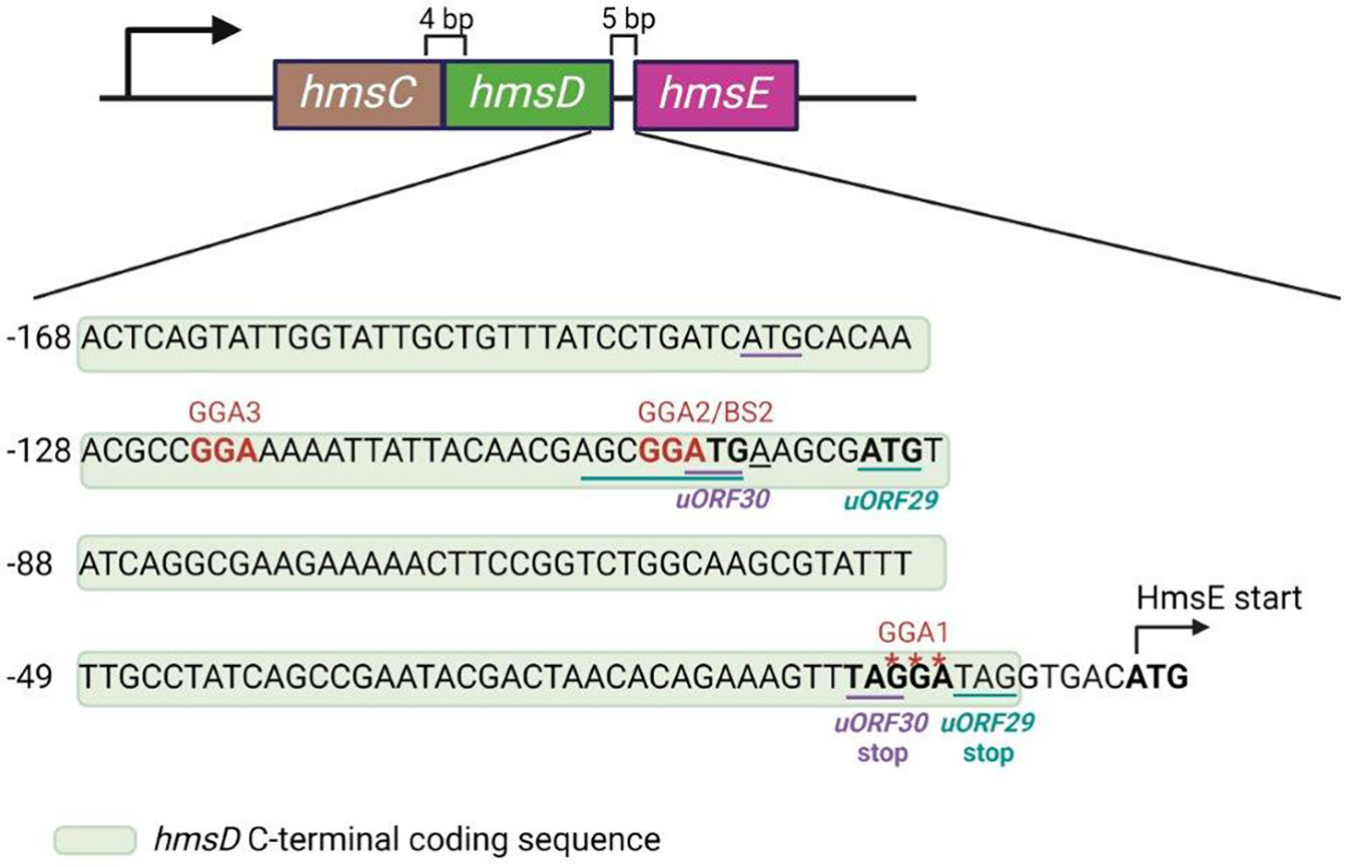
DNA sequences of the putative leader and coding sequences of the *hmsE* transcript. The *hmsE* Shine-Dalgarno (SD) and translational initiation ATG sequences are in bold font. GGA sequences are represented in red font or by red asterisks in the case of the GGA motif overlapping the *hmsE* SD sequence. Key features (start and stop codons and SD sequence) suggesting the presence of putative upstream ORFs consisting of 30 or 29 amino acids are underlined or denoted in purple and cyan, respectively. The C-terminal *hmsD* coding sequence is shaded in light green.

The region upstream (nucleotides 4147928 to 4148177) of the translational initiation codon of *hmsE* in the KIM10+ genome (GenBank AE009952.1) was examined for the presence of GGA motifs to determine if CsrA directly targets the *hmsE* mRNA. Three GGA motifs (GGA1, GGA2, and GGA3) were present within 168 nucleotides (4148011 to 4148177) of the *hmsE* ATG start codon (Fig. 1).

**FIG 2:**
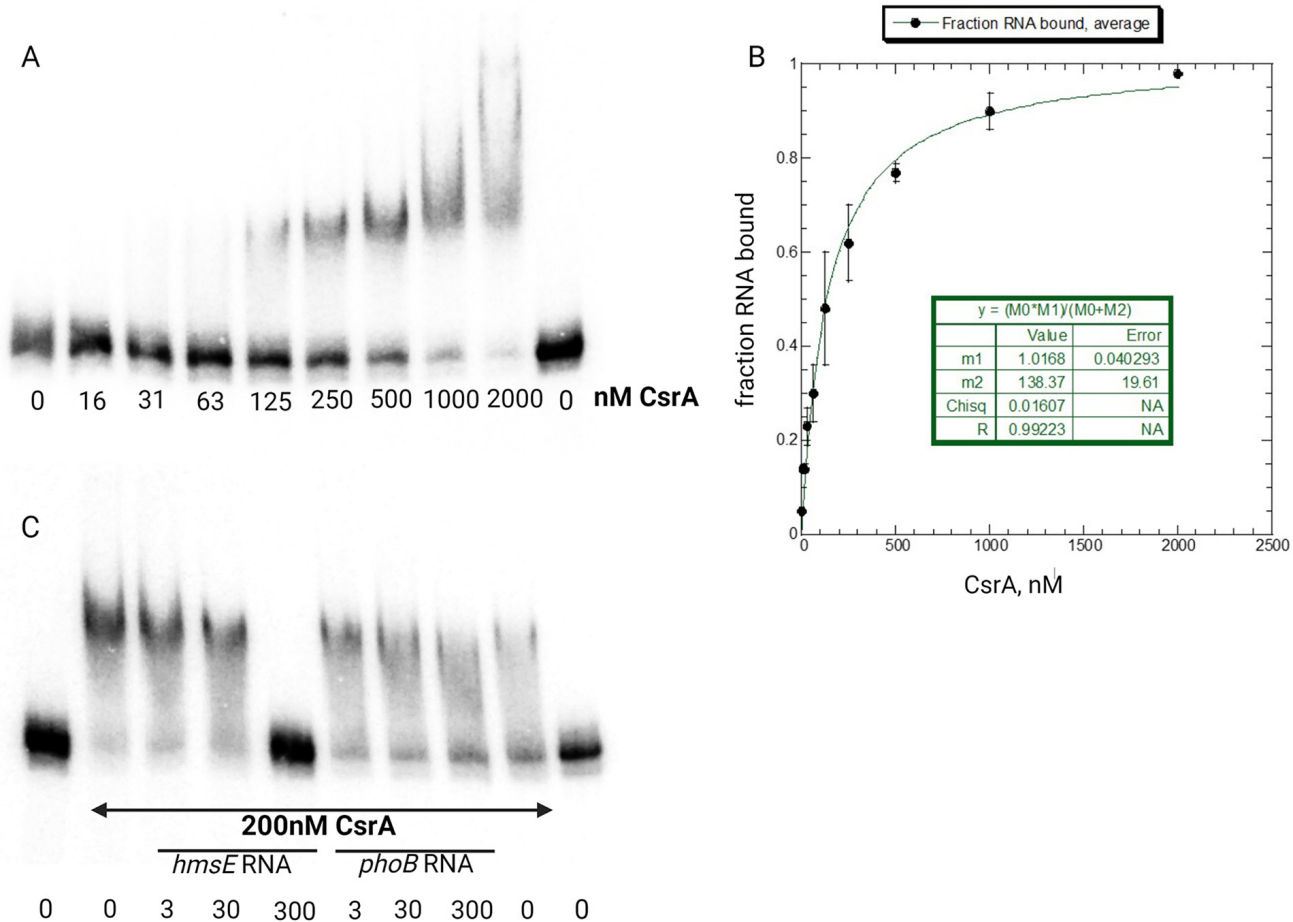
Analysis of the *hmsE* mRNA-CsrA interaction by gel mobility shift assay. (A) A labeled *hmsE* RNA at a concentration of 0.3 nM was coincubated in the presence of increasing concentrations of purified CsrA-His_6_ or with no protein. (B) Binding curve for CsrA-*hmsE* RNA interaction. The mean and error of three independent gel shifts is shown. (C) RNA competition experiment demonstrating specificity. Labeled *hmsE* RNA (0.3 nM) was incubated with CsrA-His_6_ and unlabeled specific *hmsE* RNA or nonspecific *phoB* RNA at the indicated concentrations. A representative gel of two independent experiments is shown.

Quantitative RNA gel mobility shift assays were performed to determine if CsrA binds to the *hmsE* leader region. A labeled *hmsE* transcript containing all three GGA motifs provided clear evidence of specific binding, but the resolution was insufficient to calculate a dissociation constant (*K_d_*) value, whereas binding was not observed with a transcript that contained GGA1 alone (data not shown). However, a labeled *hmsE* transcript containing GGA2 and GGA3 (−168 to −67 relative to the *hmsE* ATG start codon) shifted as a single distinct band with increasing concentrations of purified CsrA-His_6_, indicating CsrA binding (Fig. 2A). An apparent *K_d_* of 138 ± 20 nM CsrA (Fig. 2B) was determined by a nonlinear least-squares analysis, revealing that CsrA had a relatively low binding affinity for the *hmsE* transcript. To determine the specificity of the CsrA-*hmsE* RNA interaction, competitive gel shifts were performed using unlabeled specific (*hmsE*) and nonspecific (*phoB*) competitor RNAs in 10-, 100-, and 1,000-fold excess of radiolabeled *hmsE* transcript. At a concentration of 200 nM CsrA-His_6_, unlabeled *hmsE* RNA competed with the radiolabeled *hmsE* RNA for CsrA binding, while the nonspecific competitor *phoB* RNA did not, indicating that the CsrA-*hmsE* RNA interaction was specific (Fig. 2C).

### CsrA binds to one site in the *hmsE* leader.

**FIG 3:**
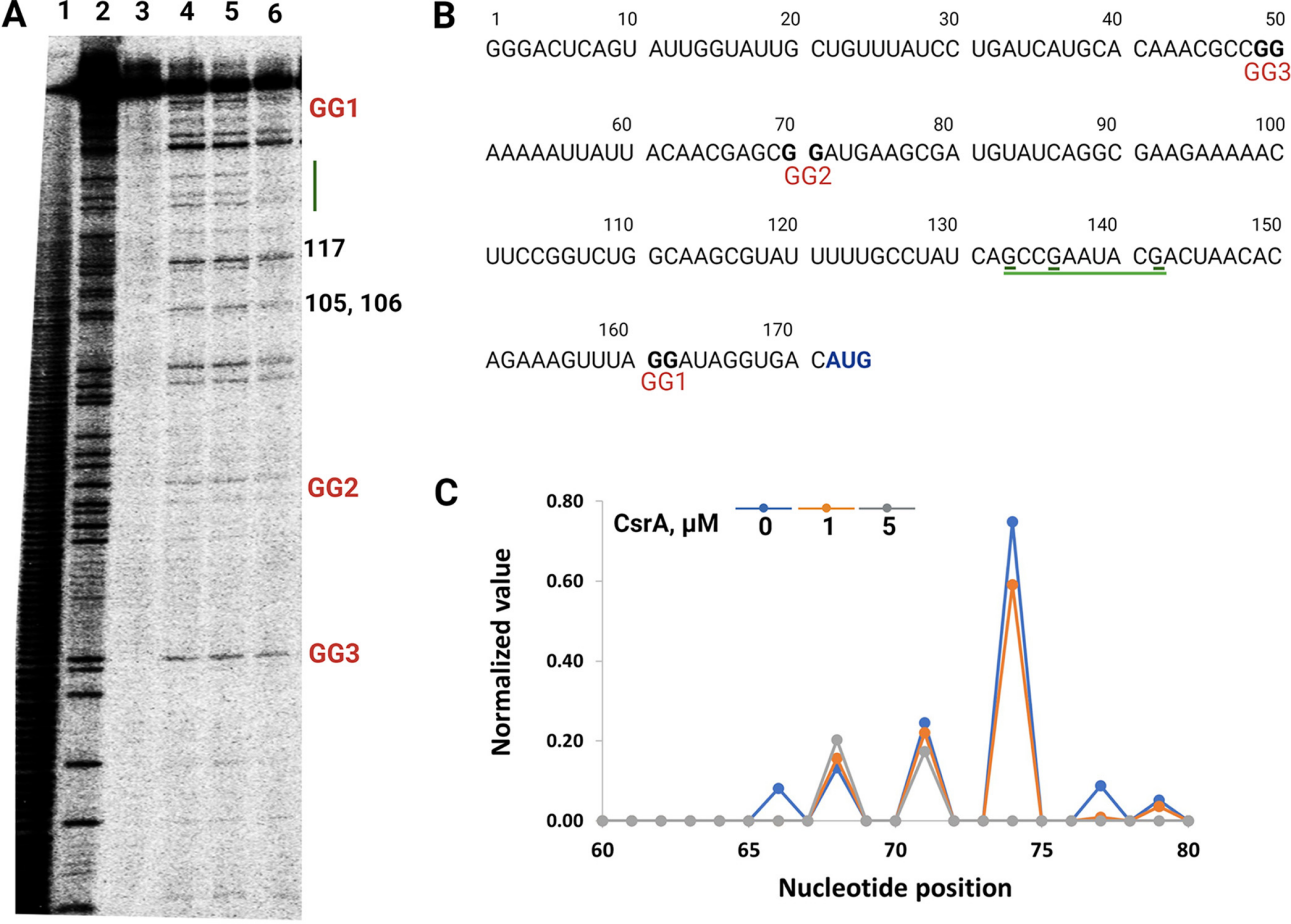
CsrA-*hmsE* RNA footprint analysis. (A) *hmsE*-mRNA footprint. Labeled *hmsE* RNA was treated with RNase T1 in the presence of increasing concentrations of CsrA. Lanes: (1) base hydrolysis ladder, every nucleotide, (2) T1 ladder, every G, (3) control without RNase T1 treatment, (4) no CsrA protein, (5) 1 μM CsrA, (6) 5 μM CsrA. The analysis was repeated three times, and a representative gel is shown. (B) RNA sequence used for the footprint analysis of CsrA. (C) A semiautomated footprint analysis (SAFA) focused on the BS2 region (G66, G71, G74, G77) showed that the G residues at G71 and G74 were protected by bound CsrA. Green lines mark other Gs that were protected by bound CsrA.

Footprint assays with a transcript of *hmsE* containing GGA1, GGA2, and GGA3 were used to identify CsrA binding sites in the *hmsE* leader region (Fig. 3). Cleavage of single-stranded G residues in the absence and presence of CsrA was probed with RNase T1. Cleavage of the second G residue of the GGA2 motif (G71), as well as G74 just downstream, was less efficient, indicating that CsrA was bound to the RNA at this site (Fig. 3A and 3C). No CsrA-dependent protection was observed for GGA1 and GGA3, implying that GGA2 is the sole binding site for CsrA in the *hmsE* transcript (Fig. 3A). In addition, the G residues at positions 125, 133, 136, and 142 were cleaved less efficiently in a CsrA-dependent manner (Fig. 3A and 3B), implying that structural changes in the mRNA occurred upon CsrA binding.

### CsrA activates *hmsE* translation by binding to GGA2.

**FIG 4:**
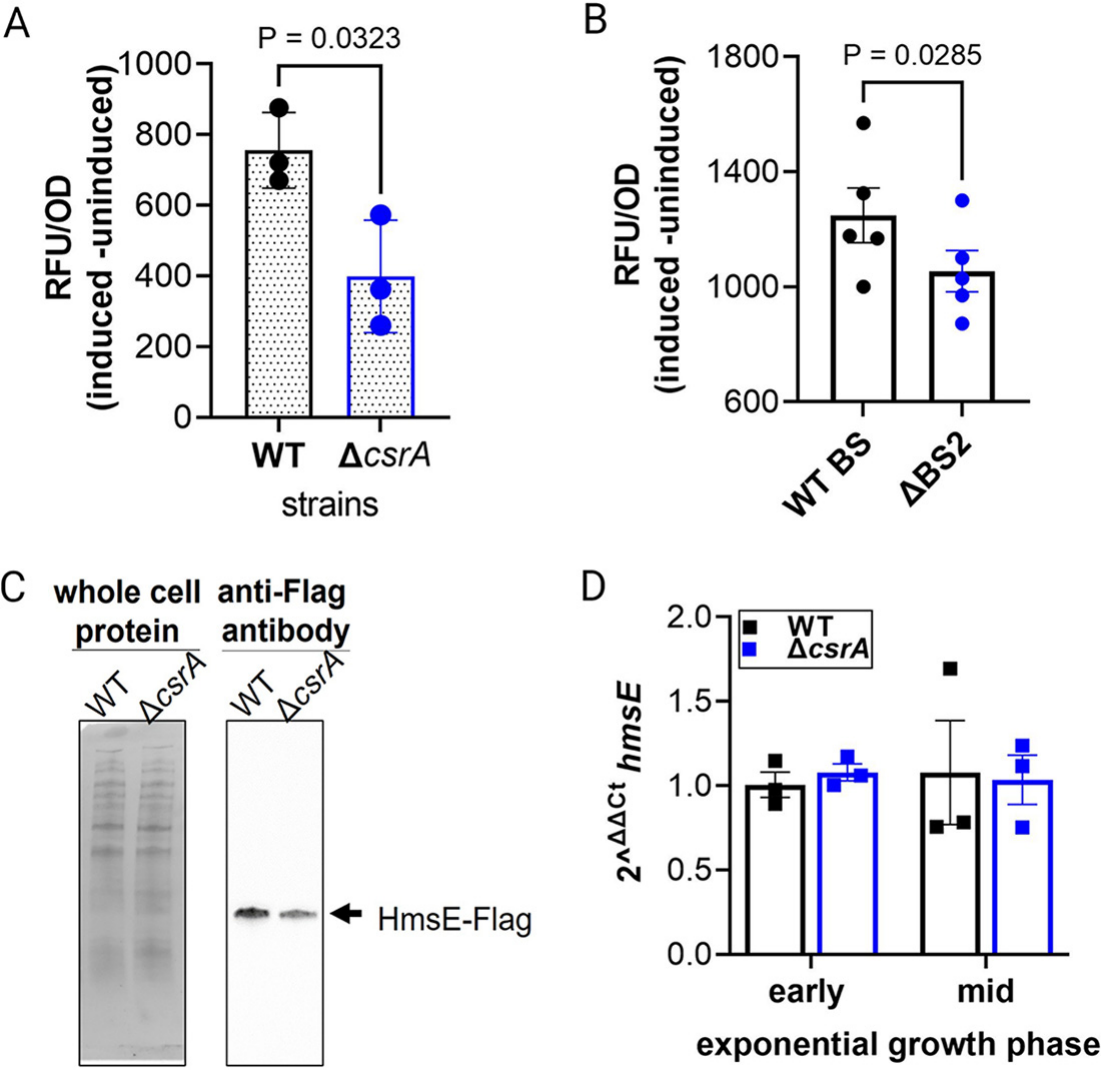
The *hmsE-gfp* expression levels are CsrA dependent. (A) The inducible *hmsE*-GFP translational fusion reporter plasmid, pMWO78::*hmsEgfp*, contains the ATc inducible promoter (PtetO), the *hmsE* 5′ leader, and 31 codons of *hmsE* fused in frame to *gfpmut3.1*. WT and Δ*csrA* strains containing the translational fusion were grown to the exponential phase, and at 3 h postinduction the relative fluorescent units (RFU) and OD_600_ were measured. The data are represented as the difference between induced and uninduced RFU/OD_600_ values. Error bars represent the mean ± standard error of the mean (SEM) of three independent experiments. An unpaired *t* test was used to determine the significance of *P* values of <0.05. (B) Inducible *hmsE*-GFP translational fusion reporter plasmids containing the WT CsrA binding site (WTBS), or a GGA→CCA mutation in GGA2 (ΔBS2) transformed into the WT strain, were assayed as described in panel A. The data are plotted as the difference between induced and uninduced RFU/OD_600_ values. Error bars represent the mean ± SEM of five independent experiments. A paired *t* test was used to determine the significance of *P* values of <0.05. (C) WT (WT_HmsE-Flag_) and Δ*csrA* (Δ*csrA*_HmsE-Flag_) strains expressing a recombinant HmsE-Flag protein were grown in TMH-gal medium. Cells were harvested at the exponential phase, and equivalent quantities of whole-cell protein lysate were resolved by SDS-PAGE (left panel) and immunoblotted with anti-Flag M2 antibody (right panel). Representative images of two biological replicates are shown. (D) WT and Δ*csrA* strains were grown to the early and mid-exponential phase in TMH-gal medium, and the steady-state abundance of *hmsE* transcripts was compared between strains. The mean ± standard deviation of three independent experiments is presented. An unpaired *t* test was used to determine significance.

Green fluorescent protein (GFP) translational fusion reporters were used to determine if translation of *hmsE* is CsrA dependent. The 5′ leader and N-terminal coding sequence (CDS) of *hmsE* was fused in frame to the *gfpmut3.1* gene. Expression of this fusion was driven by the inducible promoter, PtetO. A *csrA* mutant strain (Δ*csrA*) and the isogenic KIM6+ parental strain (wild type [WT]) transformed with the *hmsE-gfp* translational fusion were grown to the exponential phase in TMH-gal medium, and fluorescence was recorded at 3 h postinduction with anhydrotetracycline (ATc). A significant reduction in GFP expression was observed in the Δ*csrA* strain containing the *hmsE-gfp* reporter (Fig. 4A) compared to the WT strain, suggesting that translation of *hmsE* was positively regulated by CsrA.

To determine if activation of *hmsE-*GFP fusion expression was dependent on GGA2, a GG→CC mutation was introduced into the GGA2 motif and referred to as ΔBS2. In the WT strain, the *hmsE-gfp* ΔBS2 reporter showed significantly lower GFP expression levels than the WT reporter (Fig. 4B), suggesting that BS2 was involved in translational activation of *hmsE*.

Next, to determine if CsrA-mediated activation of *hmsE* expression impacted HmsE protein levels, the WT and Δ*csrA* strains were engineered with a 3×Flag epitope sequence fused in-frame to the 3′ end of the *hmsE* gene in the chromosome. These strains, named WT_HmsE-Flag_ and Δ*csrA*_HmsE-Flag_, were grown to exponential phase in TMH-gal medium and the level of HmsE-Flag protein was assessed by immunoblotting of equivalently loaded samples. The HmsE-Flag protein mean band intensity was 2.0 ± 0.3-fold greater for the WT_HmsE-Flag_ strain than for the Δ*csrA*_HmsE-Flag_ strain (Fig. 4C), indicating that HmsE protein levels were increased in the presence of CsrA.

Since translational fusions monitor expression at the level of transcription and translation, we determined whether the steady-state mRNA levels differed in WT and Δ*csrA* strains when grown to the early and mid-exponential phase. No difference in steady-state levels of the *hmsE* transcript was observed in the WT and the Δ*csrA* strains (Fig. 4D), indicating that transcript abundance of *hmsE* was unaffected by CsrA activity. Taken together with the *in vitro* binding results, our expression studies indicate that bound CsrA activates translation of *hmsE*.

### Predicted small open reading frames (ORFs) encoded in the *hmsE* leader do not affect HmsE expression.

**FIG 5:**
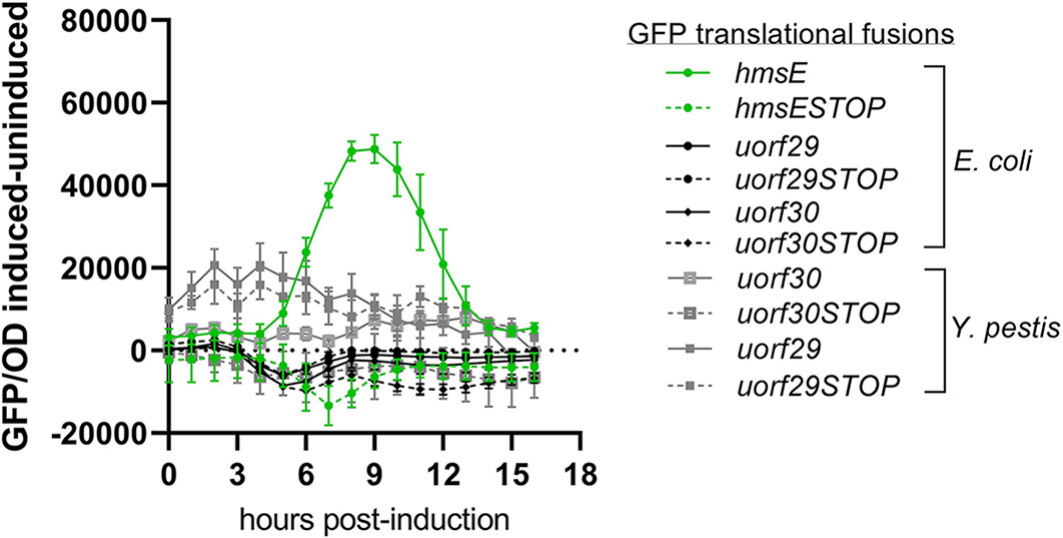
Putative small protein-coding ORFs identified in the *hmsE* leader are not expressed. Arabinose-inducible GFP expression was examined in E. coli carrying plasmids pBAD::*hmsE-gfp*, pBAD::*uORF30-gfp*, pBAD::*uORF29-gfp*, pBAD::*hmsESTOP*-*gfp*, pBAD::*uORF30STOP-gfp*, and pBAD::*UORF29STOP-gfp* grown in M9 medium (0.2% glycerol) with and without arabinose induction. Y. pestis strains carrying pBAD::*uORF30-gfp*, pBAD::*uORF29-gfp*, pBAD::*uORF30STOP-gfp*, and pBAD::*UORF29STOP-gfp* were grown in TMH-gal medium with and without arabinose induction. The data are represented as the difference between the induced and uninduced RFU/OD_600_ values. Error bars represent the mean ± SEM of three independent experiments. Black and green curves denote E. coli strains, and gray curves denote Y. pestis strains.

We identified two ORFs in the *hmsE* leader region near GGA2 (Fig. 1). One was a 30-amino acid ORF (designated uORF30; Fig. 1) with an ATG start codon that overlapped GGA2 and a UAG stop codon that overlapped the *hmsE* SD sequence. The SD sequence of the second ORF (designated uORF29; Fig. 1) appeared to overlap GGA2 with its UAG stop codon directly adjacent to the *hmsE* SD sequence. We reasoned that expression of these ORFs may obstruct ribosome binding to the *hmsE* SD sequence to reduce HmsE expression. Therefore, to determine if the putative *uORF30* and *uORF29* are translated, inducible GFP translational fusion plasmid constructs of *uORF30* and *uORF29* were generated as pBAD::*uORF30-gfp* and pBAD::*uORF29-gfp*. In addition, constructs with a stop codon replacing the second codon were also made. These constructs were named pBAD::*uORF30STOP-gfp* and pBAD::*uORF29STOP-gfp*, respectively. Similarly constructed pBAD::*hmsE-gfp* and pBAD::*hmsESTOP-gfp* served as positive controls. Expression levels of these fusions were examined following induction with arabinose. The control pBAD::*hmsESTOP-gfp* construct did not produce GFP upon induction in E. coli. No difference in expression between pBAD::*uORF30-gfp* and pBAD::*uORF30STOP-gfp* or between pBAD::*uORF29-gfp* and pBAD::*uORF29STOP-gfp* was noted in E. coli or Y. pestis, suggesting that these proteins were not expressed under these conditions (Fig. 5).

### CsrA-dependent *hmsE* mRNA translational activation promotes greater HmsD-dependent biofilm production.

**FIG 6:**
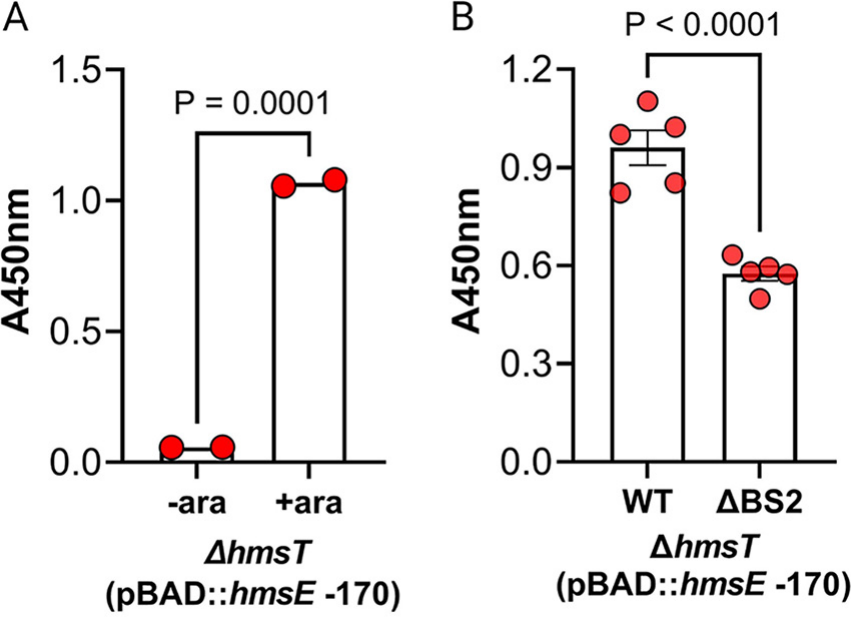
Mutation of BS2 reduces HmsD-dependent biofilm production. (A) Production of HmsD-dependent biofilm through arabinose-inducible (ara) expression of *hmsE* was confirmed in a Δ*hmsT* mutant strain. (B) Arabinose-inducible production of biofilm was compared between a Δ*hmsT* mutant strain harboring either a plasmid encoding the entire *hmsE* 5′ leader region (pBAD::*hmsE* -170) or one with a mutation in BS2 (pBAD30::*hmsE* -170 ΔBS2). Both strains were grown in TMH-gal medium and induced with 0.5% arabinose. Error bars represent the mean ± SD of two (A) or five (B) biological replicates. An unpaired *t* test was used to determine significance.

Taking advantage of an arabinose-inducible plasmid construct, pBAD::*hmsE*, that contains the *hmsE* CDS plus 170 bp of upstream leader sequence, we next asked if disruption of BS2 impacted HmsD-dependent biofilm production. Previous work demonstrated that an *hmsE* overexpression construct containing 39 bp of upstream sequence increased HmsD-dependent c-di-GMP levels and biofilm production in WT and Δ*hmsT* strains (13). Likewise, the pBAD::*hmsE* –170 construct caused increased biofilm in the Δ*hmsT* strain following induction with 0.5% arabinose (Fig. 6A). A construct with a GG→CC substitution in the GGA motif of BS2 was generated and referred to as pBAD::*hmsE* –170 ΔBS2. The plasmids were transformed into the Δ*hmsT* strain, and biofilm assays were conducted to compare HmsD-dependent biofilm formation following induction with arabinose (Fig. 6B). Compared to the Δ*hmsT* (pBAD::*hmsE* –170) strain, the Δ*hmsT* (pBAD::*hmsE* –170 ΔBS2) strain showed a significant ~2-fold decrease in biofilm formation (Fig. 6B). These results indicated that BS2 was needed to augment HmsD-dependent biofilm formation.

## DISCUSSION

Loss of function mutations that enhanced c-di-GMP-mediated biofilm formation in the flea foregut to increase transmissibility delineates the emergence of Y. pestis from its ancestor Yersinia pseudotuberculosis (25). Optimization of biofilm formation through modulation of c-di-GMP in synchrony with adaptation to the nutritional environment of the flea is also required. This process is multifaceted with regulatory checkpoints at both transcriptional (26) and posttranscriptional levels (15, 27). For example, the carbon metabolism regulator CsrA transduces alternative sugar metabolism signals to promote biofilm production in Y. pestis by translationally inhibiting the *hfq* mRNA to relieve Hfq repression of HmsT-dependent c-di-GMP biosynthesis (15). The present work demonstrates that CsrA also binds to the 5′ leader region of *hmsE* mRNA to activate its translation, which in turn leads to greater HmsD-dependent biofilm production. Because HmsD is the predominant DGC in the flea, CsrA-dependent regulation of c-di-GMP levels through HmsD may serve as an important fine-tuning mechanism for the development of a transmissible Y. pestis infection from its flea vector and spread of plague by fleabite (9, 28).

Only a few examples of CsrA-dependent activation have been identified. For example, CsrA binds to and prevents RNase E-mediated cleavage of the *flhDC* (29) and *csrB* (30) RNAs. A CsrA-mediated translation activation mechanism has been elucidated only for E. coli *ymdA* (31). In this case, CsrA binds to two sites in the *ymdA* leader region, which destabilizes a hairpin structure that otherwise sequesters the *ymdA* SD sequence. Like the *ymdA* transcript, the *hmsE* mRNA also contains a GGA motif overlapping the SD sequence, but our binding assays indicated that CsrA does not bind to this region. Instead, a site further upstream from the *hmsE* SD sequence was identified as a binding site (GGA2). Additionally, CsrA binding protects some G-residues downstream of GGA2 from RNase T1 digestion, most likely by causing RNA structural rearrangements. CsrA-dependent translational activation of *hmsE* was established using WT and BS2 mutant *hmsE-gfp* translational fusions and RNA abundance experiments. Protein expression studies substantiated these findings, as a higher level of HmsE was produced from its native locus in the WT strain than in an isogenic *csrA* mutant. Functional studies of HmsD-dependent biofilm formation using HmsE overexpression constructs containing WT or BS2 mutations served as further evidence for the role of CsrA-dependent activation of *hmsE* mRNA translation. Taken together, our results suggest a model for CsrA-dependent translational activation of *hmsE* mRNA occurring via a CsrA-induced structural change to the RNA in the *hmsE* leader region. Presumably, the restructured *hmsE* transcript is more accessible for ribosome binding.

Previous studies of the *iraD* locus of E. coli (32) determined that CsrA binds and represses translation of the short upstream *ORF27* with which *iraD* is translationally coupled, leading to translational repression of *iraD* mRNA as well. Therefore, we investigated if two *in silico* predicted putative small ORFs, named uORF30 and uORF29, present in the *hmsE* leader sequence could impact translation of *hmsE* mRNA. Under the employed testing conditions, no expression was noted from either small ORF, suggesting that these ORFs may not code for small proteins.

Homologs of *hmsCDE* have been described in pathogenic Pseudomonas aeruginosa (*yfiBNR*) and Escherichia coli CT073 (*yfiLRNB*) species, where these loci are implicated in regulating c-di-GMP and biofilm production (33–35). Whether CsrA regulates expression of these genes in P. aeruginosa and E. coli is unknown. However, the leader region of the *yfiBNR* transcript in P. aeruginosa does not contain predicted CsrA binding sites (36).

**FIG 7:**
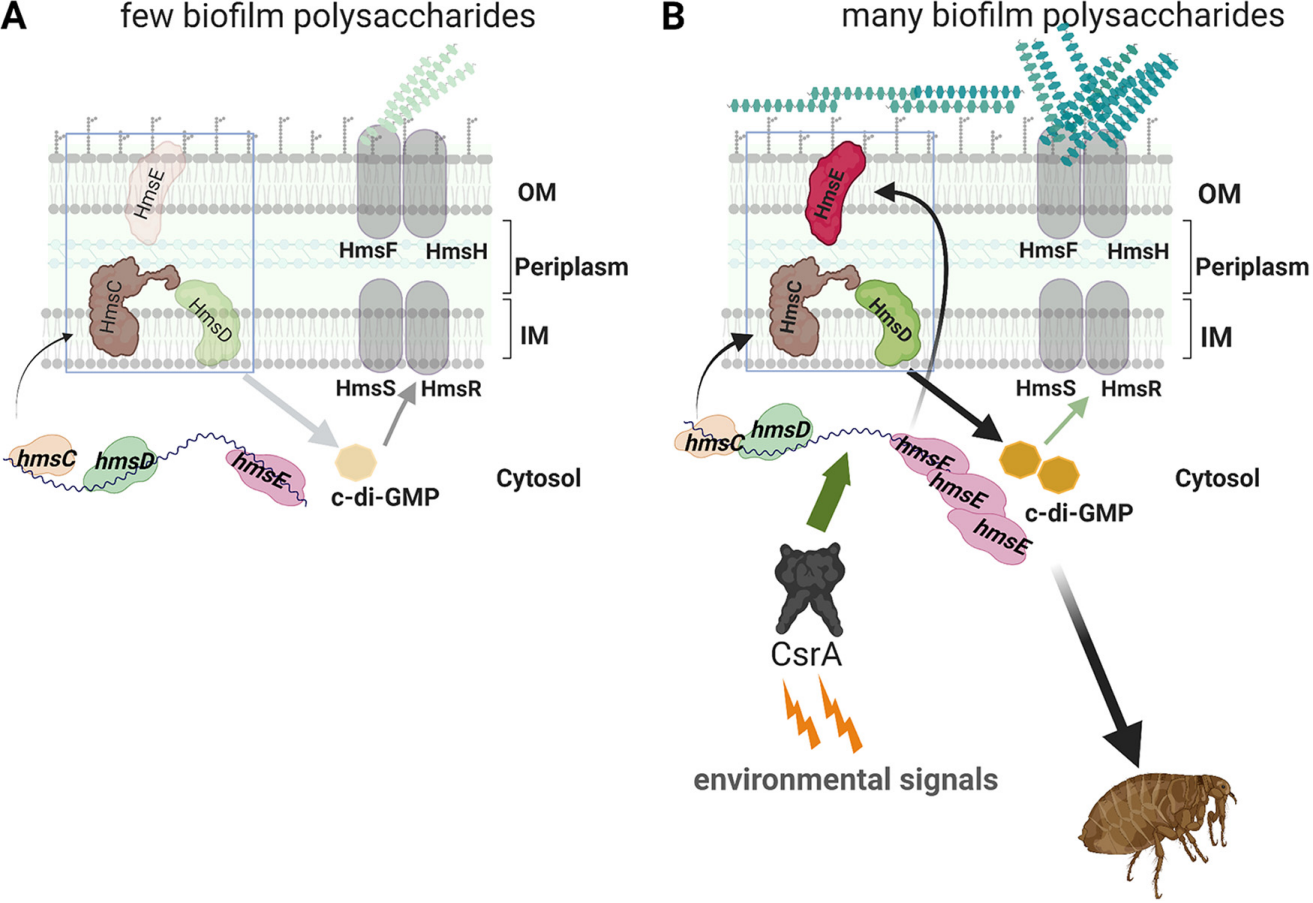
Model for regulation of Y. pestis c-di-GMP synthesis and biofilm formation through CsrA-mediated translational activation of *hmsE*. Y. pestis biofilm polysaccharides are synthesized by the gene products of the *hmsHFRS* operon, which are posttranscriptionally activated by c-di-GMP. C-di-GMP is synthesized by the HmsD diguanylate cyclase enzyme, which is inversely controlled by HmsC and HmsE. (A) Under conditions that do not favor biofilm production, HmsC inhibits HmsD DGC activity, leading to decreases in c-di-GMP production. (B) Under specific signals encountered in the flea gut, CsrA binds directly to the 5′ leader region of the *hmsE* transcript to facilitate structural alterations in the RNA such that translation is stimulated. This structural switch appears to be a predominant regulatory mechanism in the development of flea biofilm-mediated blockage. IM, inner membrane; OM, outer membrane.

The HmsD and HmsC proteins are proposed to be inner membrane-spanning proteins, with HmsC localized extensively, and HmsD partially, to the periplasm. HmsE is likely a peptidoglycan-associated lipoprotein-like outer membrane protein (12, 13). Currently, the model for how the HmsCDE tripartite signaling system operates (Fig. 7) is that modulation of the HmsC and HmsE protein levels dictate the DGC activity of HmsD. Various environmental signals differentially modulate HmsC and HmsE levels (37). A flea-specific signal likely prompts HmsE protein increases because an *hmsE* mutant shows poor biofilm-mediated blockage of fleas but wild-type biofilm levels *in vitro* (13). A reducing environment in the periplasm facilitates decreases in HmsC, but not HmsE, levels (14, 37). Consistent with the model, this study demonstrates that CsrA increases HmsE expression and enhancement of HmsD-dependent biofilm production; however, the flea-specific conditional determinants for this interaction remain elusive.

Although CsrA appears to stimulate c-di-GMP levels through activation of HmsT and HmsD activity, the spatial and temporal contexts of both DGC activities during Y. pestis biofilm-mediated foregut blockage is unknown. Certainly, spatially localized cytosolic pools of c-di-GMP within the cell that are distinctly contributed to by HmsT or HmsD activity may need to be individually tuned to maximize biofilm production with changing environmental conditions. Such knowledge may explain the disparate roles of HmsT and HmsD during flea blockage. Nevertheless, in this study we show that like in other bacteria, CsrA controls cellular c-di-GMP pools through more than one mechanism, reiterating that CsrA is a significant mediator that coordinates biofilm production in accordance with the bacterial nutritional environment. This study also serves as further evidence that a highly evolved regulatory network in c-di-GMP synthesis supports the regurgitative transmission of Y. pestis by fleabite.

## MATERIALS AND METHODS

### Bacterial strains, growth conditions, and plasmids.

Bacterial strains are listed in Table 1. The Y. pestis KIM6+ parental strain was designated as the wild-type (WT) strain in this study. All Y. pestis strains were routinely cultured on heart infusion broth (HIB) and on Congo red heart infusion agar (38) to verify the biofilm status of the strains. Strains grown in broth cultures were aerated continuously at 26°C. To support robust levels of biofilm, the chemically defined TMH medium (39) supplemented with 0.2% galactose (TMH-gal) was used as previously described (15). E. coli strains were generally cultured in LB medium, except for translational fusion reporter studies where M9 minimal medium supplemented with 0.2% glycerol was used. The primers are listed in Table 2. All plasmid constructs were verified by DNA sequencing (Eurofins Genomics or Azenta Life Sciences).

**TABLE 1:** Strains and plasmids used in this study

Strain or plasmid	Description^*a*^	Reference or source
Y. pestis KIM6+ strains		
WT	Pgm+ pCD1- pMT1+ pPCP1+, parental strain	15
Δ*csrA*	Δ*csrA* 4:12	16
Δ*csrA*::*csrA*	Δ*csrA* 4:12 (*att*Tn7::*csrA*)	15
WT *hmsE-gfp*	WT pMWO78::*hmsE-gfp*	This study
Δ*csrA hmsE-gfp*	Δ*csrA* pMWO78::*hmsE-gfp*	This study
WT *hmsE-gfp* ΔBS2	WT with pMWO78::*hmsE-gfp* ΔBS2	This study
WT_HmsE-Flag_	WT with a 3×Flag sequence tag at the 3′ end of the *hmsE* gene	This study
Δ*csrA*_HmsE-Flag_	Δ*csrA* with a 3×Flag sequence tag at the 3′ end of the *hmsE* gene	This study
WT pBAD::*hmsE-gfp*	WT with pBAD::*hmsE-gfp*	This study
WT pBAD::*uORF30-gfp*	WT with pBAD::*uORF30-gfp*	This study
WT pBAD::uORF30STOP*-gfp*	WT with pBAD::uORF30STOP*-gfp*	This study
WT pBAD::*uORF29-gfp*	WT with pBAD::*uORF*29-*gfp*	This study
WT pBAD::uORF29STOP*-gfp*	WT with pBAD::uORF29STOP*-gfp*	This study
Δ*hmsT* (pBAD::*hmsE* -170)	Δ*hmsT* strain transformed with pBAD::*hmsE*	This study
Δ*hmsT* (pBAD::*hmsE* -170 ΔBS2)	Δ*hmsT* strain transformed with pBAD::*hmsE* ΔBS2	This study
E. coli Bl21 (DE3) pLys transformed with plasmids		
pET28A::*csrA-his_6_*		15
pBAD::*hmsE-gfp*		This study
pBAD::*hmsESTOP-gfp*		This study
pBAD::*uORF30-gfp*		This study
pBAD::*uORF30STOP-gfp*		This study
pBAD::*uORF29-gfp*		This study
pBAD::*uORF29STOP-gfp*		This study
Plasmids		
pFU34	Amp^r^; source of *gfpmut3.1* used for reporter fusions	41
pMWO78	Spec^r^; p15A; contains the *tet* operator/promoter system	42
pBAD30	Amp^r^; p15a; pBAD arabinose inducible promoter	49
pMWO78::*hmsE-gfp*	Spec^r^; −170 bp, +93 bp of *hmsE* 5′ UTR plus *hmsE* CDS	This study
pMWO78::*hmsE-gfp* ΔBS2	Spec^r^; −170 bp, +93 bp of *hmsE* 5′ UTR plus *hmsE* CDS, BS2 GGA→CCA	This study
pBAD::*hmsE-gfp*	Amp^r^; −170 bp, +93 bp of *hmsE* 5′ UTR plus *hmsE* CDS	This study
pBAD::*uORF30-gfp*	Amp^r^; −167 bp, −14 bp of *hmsE* 5′ UTR	This study
pBAD::*uORF30STOP-gfp*	Amp^r^; −167 bp to −14 bp of *hmsE* 5′ UTR, second codon of uORF30 changed from AAG to TAG	This study
pBAD::*uORF29-gfp*	Amp^r^; −167 bp to −9 bp of *hmsE* 5′ UTR,	This study
pBAD::*uORF29STOP-gfp*	Amp^r^; −167 bp to −9 bp of *hmsE* 5′ UTR, 2nd codon of uORF29 changed from ATG to TAG	This study
pBAD::*hmsE -170*	Amp^r^; 170 bp upstream plus *hmsE* CDS	This study
pBAD::*hmsE* -170 ΔBS2	Amp^r^; 170 bp upstream plus *hmsE* CDS; BS2 GGA→CCA SDM	This study
pBAD::*hmsESTOP-gfp*	Amp^r^, 167 bp upstream plus *hmsE* CDS to +93 where 2nd codon of *hmsE* changed from CAG to TAG	This study

a UTR, untranslated region.

**TABLE 2:** Oligonucleotides used in this study

Description	Sequence	Source of reference
T7 hmsE for1 (174 nt and 104 nt RNA)	GTAATACGACTCACTATAGGGACTCAGTATTGGTATTGCTGTTTATC	This study
hmsE REV1 (174 nt RNA)	CATGTCACCTATCCTAAACTTTCTGTG	This study
hmsE REV2	GGAAGTTTTTCTTCGCCTGATACATCGC	This study
T7 hmsE for2 (91 nt RNA)	GTAATACGACTCACTATAGGGTCTGGCAAGCGTATTTTTGCCTATC	This study
hmsE REV3	CGCTTGTTTTGCATTTTCTGCATGTC	This study
p496, qRT-PCR *hmsE* F	TTGGCCGGATATTGTCAGATGTGG	This study
p497, qRT-PCR *hmsE* R	AATACCGACGGTAGCGAGAGTATC	This study
p1033, fusion 5′ UTR *hmsE*	GATCGGGAATTCACTCAGTATTGGTATTGCTG	This study
p1035, fusion 3′ *hmsE*	AAAAGTTCTTCTCCTTTACGCATTTTAGCCTGACACCCCACTAATAG	This study
p1036, fusion 5′ *gfp* for *hmsE* (31 codons)	CTATTAGTGGGGTGTCAGGCTAAAATGCGTAAAGGAGAAGAACTTTTCAC	This study
p126, fusion 3′ *gfp*	TTATTTGTATAGTTCATCCATGCCATGTGTAATCC	15
p1146, SDM hmsE_BS2_CCA_F	GAAAAATTATTACAACGAGCCCATGAAGCGATGTATCAGGCG	This study
p1147, SDM hmsE_BS2_CCA_R	CGCCTGATACATCGCTTCATGGGCTCGTTGTAATAATTTTTC	This study
p1097 T7 prom + 5′ *hmsE*	GTAATACGACTCACTATAGGGCTCAGTATTGGTATTGCTGTTTATC	This study
p1081, pSUB11f	GACTACAAAGACCATGACGG	This study
p1082, pSUB11r	GGTCCATATGAATATCCTCCTTAG	This study
p1090 5′ hmsE-3×Flag F (upstream of *hmsE* 3′ end)	GTTTAGGATAGGTGACATGCAG	This study
p1091, 5′ hmsE-3×Flag R (upstream of *hmsE* 3′ end)	CCGTCATGGTCTTTGTAGTCGGACGCGGTGATAATAATGG	This study
P1092, 3′ hmsE-3×Flag F (downstream of *hmsE* 3′ end)	CTAAGGAGGATATTCATATGGACCCGTCCTTTATTTACCGGTTTG	This study
p1093, 3′ hmsE-3×Flag R (downstream of *hmsE* 3′ end)	CGCATAAACTTCATCCAGAC	This study
p1157	ATGCGTAAAGGAGAAGAACTTTTCACTGGAGTTG	This study
p1158	TCCTAAACTTTCTGTGTTAGTCGTATTCGGCTG	This study
p1159, hmsE90bpLP_STOP F	TTACAACGAGCGGATGTAGCGATGTATCAGGCG	This study
p1160, hmsE90bpLP_STOP R	CGCCTGATACATCGCTACATCCGCTCGTTGTAA	This study
p1164	AACTTTCTGTGTTAGTCGTATTCGGC	This study
p1165	GGATGAAGCGATGTAGCAGGCGAAGAAAAAC	This study
p1166	GTTTTTCTTCGCCTGCTACATCGCTTCATCC	This study
p1175, hmsE_STOP_F	GTTTAGGATAGGTGACATGTAGAAAATGCAAAACAAGCG	This study
p1176, hmsE_STOP_R	CGCTTGTTTTGCATTTTCTaCATGTCACCTATCCTAAAC	This study

### Quantitative reverse transcription-PCR (qRT-PCR) assays.

Early and mid-exponential phase TMH-gal cultures of strains of interest were mixed with RNAprotect bacterial reagent (Qiagen). Cells were then harvested at room temperature and stored at −80°C until RNA isolation. RNA isolation and qRT-PCR were conducted as previously described (15, 40).

### GFP translation fusion reporter construction and assays.

The GFP translation fusion reporter using the pMW078 plasmid with the anhydrotetracycline (ATc) inducible promoter was made and assayed as previously described (15). The upstream 5′ leader and first 31 codons of the *hmsE* gene were amplified by PCR from the genomic DNA of the WT strain with primer pair p1033/p1035. The generated fragment was then spliced by overlap extension PCR (SOE-PCR) to the *gfpmut3.1* sequence from pFU34 that was generated with primer pair p1036/p126 (41). The resulting amplification product was digested with EcoRI and cloned into pMWO78 (42) at the EcoRI*/*SmaI sites and named pMWO78::*hmsE-gfp*.

To generate a reporter construct with a single mutation in GGA2, site-directed mutagenesis (SDM) PCR was performed on plasmid pMWO78::*hmsE-gfp* with primer pair p1146/p1147 (Table 1). This plasmid was named pMWO78::*hmsE-gfp* ΔBS2.

Arabinose-inducible GFP translational fusion reporter constructs of *uORF30* (LP90) and *uORF29* (LP87) were generated in pBAD30. First, the pBAD::*hmsE-gfp* construct was made using pMWO78::*hmsE-gfp* as a template for amplification of the *hmsE-gfp* DNA sequence using primer pair p1097/p126. This fragment was cloned into the SmaI site of pBAD30. The pBAD::*hmsE-gfp* was then used as a template for SDM with primers p1175/p1176 to generate pBAD::*hmsESTOP*-*gfp*, in which the second codon of *hmsE* was changed to a stop codon. Next, to generate *uORF30-gfp* and *uORF29-gfp* reporter constructs, inverse PCR with primers p1164/p1157 and p1157/p1158, respectively was performed on the pBAD::*hmsE-gfp* plasmid template. The amplified fragments were ligated to generate pBAD::*uORF30-gfp* and pBAD::*uORF29-gfp.* Finally, to eliminate expression of *uORF30-gfp* and *uORF29-gfp*, the second codon was changed to a stop codon in plasmids pBAD::*uORF30-gfp* and pBAD::*uORF29-gfp* with primer pairs p1159/p1160 and p1165/p1166, respectively, using SDM. The resulting plasmids were named pBAD::*uORF30STOP-gfp* and pBAD::*uORF29STOP-gfp.*Plasmids were transformed into Escherichia coli or Y. pestis strains (Tables 1 and 2).

### Construction of arabinose-inducible *hmsE*-overexpressing strains.

Using the PCR primer pair p1033/p1093, the *hmsE* 5′ leader region and the coding sequence (CDS) were amplified from WT genomic DNA. This amplified fragment was digested with EcoRI and cloned into the EcoRI*/*SmaI sites of pBAD30 to generate the pBAD::*hmsE* plasmid. SDM PCR was performed on the pBAD::*hmsE* plasmid with primer pair p1146/p1147 to generate a pBAD::*hmsE* construct with a single mutation in CsrA binding site 2 (BS2), named pBAD::*hmsE* –170 ΔBS2. Plasmid constructs were transformed into the Y. pestis Δ*hmsT* mutant strain.

### CsrA-His_6_ purification.

Purified CsrA-His_6_ was obtained from the induced BL21λDE3 pLysS pET28A::*csrA-his_6_* strain and subjected to expression and purification as previously described (15, 43). Exceptions were that dialysis was accomplished using a 7,000 molecular weight cutoff (MWCO) Slide-A-Lyzer G2 dialysis cassette (Thermo Fisher Scientific) with 100 mM Tris-HCl, 100 mM NaCl, and 10% glycerol, pH 7.5, as the dialysis buffer, and a follow-up buffer exchange step was omitted.

### RNA gel mobility shift assay.

Quantitative gel mobility shift assays followed a published procedure (44). Three different-sized RNAs were synthesized with the RNAMaxx kit (Agilent Technologies) using PCR-generated DNA templates: 174 nucleotides [nt]; (−168 to +3 relative to the *hmsE* translational start codon) with all 3 GGA repeats, 104 nt (−168 to −67 relative to the *hmsE* translational start codon) with GGA2 and 3, and 91 nt (−67 to +23 relative to the *hmsE* translational start codon) with GGA1. The template for PCR was the pBAD30::*hmsE* -170 plasmid. Gel-purified RNAs were dephosphorylated and then 5′ end labeled using T4 polynucleotide kinase (New England Biolabs) and [γ-^32^P]ATP. Labeled RNAs were renatured by heating for 1 min at 90°C, followed by slow cooling at room temperature. Binding reaction mixtures (10 μL) contained 0.1 to 0.3 nM labeled RNA, 10 mM Tris-HCl (pH 7.5), 10 mM MgCl_2_, 100 mM KCl, 40 ng of yeast tRNA, 7.5% glycerol, 0.1 mg/mL of xylene cyanol, and various concentrations of purified CsrA-His_6_. Competition assays also contained unlabeled RNA competitors. Reaction mixtures were incubated for 30 min at 37°C to allow CsrA-RNA complex formation, and then samples were fractionated through 8 to 12.5% nondenaturing polyacrylamide gels (19:1 ratio) using 0.5× Tris-borate-EDTA (TBE) buffer. Free and bound RNA species were visualized with a Typhoon 8600 variable-mode phosphorimager (GE Healthcare Life Sciences). CsrA-RNA interaction was quantified as described previously (44).

### RNase T1 footprint assays.

CsrA-*hmsE* RNA footprint assays followed a published procedure (44). The 174-nt RNA (−168 to +3 relative to the *hmsE* translational start codon) with all 3 GGA repeats was labeled as described above for the gel mobility shift assay. The reaction mixtures were identical to those in the gel shift assay except that the concentration of labeled RNA was raised to 2 nM, and 1 μg of acetylated bovine serum albumin (BSA) was added to each reaction mixture. Reaction mixtures were incubated for 30 min at 37°C to allow CsrA-RNA complex formation, and then RNase T1 (0.08 U) was added, and incubation was continued for 15 min at 37°C. Reactions were stopped by adding 10 μL of gel loading buffer (95% formamide, 0.025% sodium dodecyl sulfate [SDS], 20 mM EDTA, 0.025% bromophenol blue, 0.025% xylene cyanol). Samples were heated for 5 min at 90°C and then fractionated through 6% sequencing gels. Cleavage patterns were examined using a phosphorimager and quantified using semiautomated footprinting analysis software (SAFA) (45). Each number was normalized to the 95th percentile value of the column.

### Construction of HmsE-3×Flag-expressing strains.

The WT_HmsE-Flag_ and Δ*csrA*_HmsE-Flag_ strains expressing recombinant HmsE protein with a 3×Flag epitope tag at the C terminus (HmsE-Flag) were constructed by replacing the native chromosomal gene using a modification of the lambda red recombination system (46). To generate these strains, a 520-bp upstream (primers p1090/p1091) region and a 509-bp downstream (primers p1092/p1093) region of the 3′ end of the *hmsE* gene were amplified separately. Splice-overlap PCR joined these fragments to another PCR amplicon containing a kanamycin resistance-encoding gene and the 3 × Flag epitope tag sequence that was previously amplified (primers p1081/p1082) from pSUB11. The resultant product was transformed into WT and Δ*csrA* competent cells expressing the lambda red recombinase. Kanamycin-resistant transformants were confirmed by PCR to contain the insertion. The kanamycin resistance cassette was excised as previously described (47), and DNA sequencing verified the intended gene replacement.

### Anti-FLAG tag immunoblotting.

For immunoblotting, the WT_HmsE-Flag_ and Δ*csrA*_HmsE-Flag_ strains were cultured in TMH-gal medium, and cells were harvested during the exponential growth phase. Cells were suspended and boiled in 1× Laemmli sample buffer for 5 min. Lysates were separated by SDS page and transferred to a 0.2- μm nitrocellulose membrane (Bio-Rad). HmsE-Flag protein was detected using monoclonal anti-FLAG M2 (Sigma) antibodies (1:20,000), affinity purified horseradish peroxidase (HRP)-labeled goat anti-mouse (KPL) antibodies (1:10,000), and the SuperSignal West Femto substrate (Thermo Scientific). The No-Stain protein labeling reagent (Invitrogen) was used to normalize total protein loaded per lane of the gel. The HmsE-Flag protein was quantified by densitometry using the ChemiDoc software Image Lab 4.1 (Bio-Rad).

### Arabinose-inducible GFP translational fusion assays.

Overnight HIB cultures of Y. pestis strains harboring pBAD plasmid constructs were diluted 1:100 in TMH-gal. Overnight LB cultures of E. coli strains harboring pBAD plasmids were diluted 1:100 in N-minimal medium supplemented with 0.2% glycerol. The 1:100 dilutions were split in two with, one receiving 0.2% l-arabinose. Triplicate aliquots of 100 μL were added to a 96-well uClear black plate, and the plate was shaken in the Tecan Spark device for 16 h, during which GFP and optical density at 600 nm (OD_600_) readings were taken every hour.

### Biofilm assays.

Overnight HIB cultures of the Δ*hmsT* Y. pestis strain harboring pBAD plasmid constructs were diluted 1:100 in TMH-gal medium and induced with 0.5% l-arabinose. Triplicate aliquots of 100 μL were added to a 96-well plate and the plate was shaken for 24 h at room temperature. Biofilm was quantified by safranin staining as previously described (48).

### Statistical analysis.

Details of statistical analysis using GraphPad Prism 9.4.1 are provided in the legends of Fig. 4 to 6.

### Data availability.

Data are available on reasonable request from the corresponding author.
